# Response of pediatric uveitis to TNFα inhibitors

**DOI:** 10.1186/1546-0096-10-S1-A79

**Published:** 2012-07-13

**Authors:** Melissa A Lerman, Monte Mills, Marshall Joffe, Terri H Finkel, Sean Hennessy, John H Kempen

**Affiliations:** 1 The Children's Hospital of Phiadelphia, Philadelphia, PA, USA; 2 University of Pennsylvania School of Medicine, Philadelphia, PA, USA

## Purpose

Non-infectious uveitis (“uveitis”) is an important cause of visual loss amongst children. Although TNFα inhibitors (anti-TNFα) are increasingly used to treat pediatric uveitis, the outcomes of treatment with these agents have been poorly described. We are studying the largest, and only multicenter, group of patients with pediatric uveitis in order to more precisely estimate the outcomes of treatment with anti-TNFα and to evaluate which factors increase the likelihood of achieving uveitis quiescence after treatment. The final cohort will include subjects from the five-center Systemic Immunosuppressive Therapy for Eye Diseases Cohort Study, The Children’s Hospital of Philadelphia (CHOP), and Hershey Medical Center. Here we report preliminary results from subjects at CHOP.

## Methods

This cohort included a child if (s)he was <19 years old at treatment outset, was treated with his/her first ever anti-TNFα while having active uveitis, and had uveitis activity information within 15 days of starting the drug. A child could also be treated with steroids and/or methotrexate. Active uveitis was defined according to the SUN Working Group (2005). Quiescence was defined as having inactive uveitis in both eyes at sequential ophthalmologic exams ≥28 days apart while no longer receiving steroids. Survival analysis evaluated time to quiescence.

## Results

Of the 109 children treated for uveitis at CHOP, 39 have been analyzed who were treated with anti-TNFα. Eighteen met inclusion criteria and had adequate follow-up information. Of these, 67% were female, 56% were Caucasian, 23% were diagnosed <6 years, 33% had juvenile idiopathic arthritis (JIA), 11% had oligo-articular JIA, 47% had painful uveitis, and 22% were ANA positive. All of those who were ANA positive were female. While there was a statistically significant association between female sex and JIA (p=0.034) there was not an association between ANA positivity and oligo-articular JIA (p=0.32) or male gender and painful uveitis (p=0.84). The total analysis time was 20.8 years (0.3-4.3 years/patient). The probability of a child achieving quiescence was 0 at 3 months; 0.33 at 6 months (95% confidence interval 0.27, 0.73); and 0.47 at 12 months (0.23, 0.73). Fifty percent of subjects achieved remission by 13 months (6.3, 19.4). Ultimately, 85% of the cohort achieved quiescence. Achievement of success was not associated with sex, race, age <6 years at diagnosis, JIA, oligoarticular JIA, painful uveitis, or ANA status. Because 16/18 children were treated with infliximab, the impact of anti-TNFα type was not explored.

## Conclusion

In this preliminary study, treatment with a first-ever anti-TNFα resulted in quiescence in 47% of subjects at 12 months. This rate is slower than might be expected based on other studies. This may reflect our more stringent definition of remission. Other studies have used achievement of uveitis inactivity alone or inactivity with a decrease in steroid as outcome measures; we defined quiescence as withdrawal of all steroids. Given the limited sample size, we were unable to identify independent factors associated with quiescence. We plan to identify associated factors and develop an explanatory model of success with our final cohort of >100 patients.

## Disclosure

Melissa A. Lerman: None; Monte Mills: None; Marshall Joffe: None; Terri H. Finkel: None; Sean Hennessy: None; John H. Kempen: None.

